# Indoor Navigation for People With Visual Impairment in Canada: Participatory Co-Design and Interdisciplinary Study of the Edge A-Eye Platform

**DOI:** 10.2196/81347

**Published:** 2026-07-31

**Authors:** Soutongnoma Safiata Kaboré, Deborah Annan, Prajjol Raj Puri, Sergio Mejia Romero, Nathalie Gingras‑Royer, Justin Gauthier, Hồng Nhung, Phuong Anh Nguyen, Benoit Pelletier, Kim Khoa Nguyen, Hassane Alami, Joseph Paul Nemargut

**Affiliations:** 1 School of Optomtery, University of Montreal, 3744 Jean-Brillant Street, Montreal, QC, H3T 1P1, Canada, 1 5145766589; 2 Department of Electrical Engineering, School of Higher Technology, Montreal, QC, Canada; 3 Public Health School, University of Montreal, Montreal, QC, Canada

**Keywords:** visual impairment, indoor navigation, AI-based assistive technology, co-design, accessibility, human-centered design, universal design, Canada, artificial intelligence, mobile phone

## Abstract

**Background:**

Indoor navigation remains a major challenge for people with visual impairments, affecting autonomy, safety, and quality of life. While navigation‑based assistive technologies leveraging artificial intelligence and mobile computing show promise, their adoption remains limited due to insufficient user-centered and context-aware design.

**Objective:**

This study aimed to co-design Edge A-Eye, an inclusive artificial intelligence–powered indoor navigation platform for people with visual impairment in Canada, using a participatory and interdisciplinary approach grounded in accessibility, rehabilitation, and human-centered design principles.

**Methods:**

From May 2024 to December 2025, we conducted research using an 8-step iterative co-design process. A total of 12 people with visual impairment and 2 specialists in vision rehabilitation participated in bilingual focus groups and semistructured interviews. A co-creation workshop gathered 21 stakeholders (users, clinicians, researchers, and industry partners). Successive prototypes were evaluated in controlled environments and real-world public settings. Data were analyzed thematically using an inductive-deductive approach informed by human-centered and inclusive design frameworks.

**Results:**

Continuous stakeholder engagement reinforced the relevance and usability of the evolving prototypes. Participant feedback guided major design decisions, such as optimizing iPhone (Apple Inc) compatibility, excluding the gyroscope due to usability and battery constraints, and integrating hands-free navigation. The process also addressed cross-cutting issues such as digital accessibility, cybersecurity, universal design, and equitable access for users with varying technological literacy.

**Conclusions:**

The Edge A-Eye project demonstrates how a sustained participatory co-design approach can meaningfully shape assistive technology innovation. By integrating the expertise of people with visual impairment, clinicians, researchers, and industry partners at every stage, the resulting platform is better aligned with real-world needs, safety considerations, and user expectations. This work provides a replicable framework for developing inclusive, context-aware indoor navigation technologies.

## Introduction

### Global Context

More than 2.2 billion people have impaired near or distance vision around the globe, and this number is projected to double by 2050 [1]. Visual impairment affects people of all ages, but most of those affected are older than 50 years [2]. In 2017, over 1,519,000 Canadians reported having a visual disability, accounting for 5.4% of the Canadian population [3]. These individuals encounter multiple daily barriers, including physical and functional obstacles that hinder their full participation in society [4]. The most frequently mentioned obstacles include reading and accessing written information [5], engaging in leisure activities and shopping [5], as well as using public transport [6]. Visual impairment imposes a significant burden globally, not only in human and social terms but also in relation to health and economic costs, with annual productivity losses estimated at US $411 billion [7]. As the global population continues to age and the prevalence of noncommunicable diseases rises, it is estimated that by 2050, a total of 3.5 billion people will require access to assistive technologies (ATs) [8].

### Importance of Navigation-Based AT

Navigating within an environment is a fundamental aspect of the human experience, enabling individuals to carry out daily activities and engage with their surroundings [9]. Navigation-based assistive technologies (NATs) are crucial for enhancing the functional abilities of people with visual impairments [10]. NATs play a vital role in maintaining or improving the functional capabilities of individuals with disabilities and significantly impact their quality of life [11]. NATs have proliferated in recent decades to enhance orientation and mobility (O&M) for individuals with blindness and low-vision [12]. These technologies are incredibly diverse, ranging from prototypes to commercially available solutions, including electronic travel aids, smartphone apps, sensing systems, wearables, Internet of Things devices, and artificial intelligence (AI)–powered assistants [12]. O&M specialists typically provide vision rehabilitation services, instructing individuals with little to no vision on the use of NATs to support independent travel [13]. These technologies can function independently or in conjunction with traditional aids such as white canes and guide dogs [10], providing spatial awareness, obstacle detection, situational guidance, and real-time information for both indoor and outdoor navigation [14]. Ideally, NATs are designed to support and enhance existing adaptive skills, including cognitive mapping, the interpretation of nonvisual cues, and familiar travel strategies [15]. As highlighted by Nemargut, O&M training equips people with visual impairments with the foundational skills needed to interpret spatial cues, build cognitive maps, and navigate safely and independently across diverse environments [16]. This underscores that the effectiveness of NATs relies not only on technological design but also on users’ existing O&M competencies, which fundamentally shape how assistive tools are interpreted and applied in real-world contexts. Although important progress has been made in the development of AT for people with visual impairment, many devices continue to endure design approaches that do not adequately reflect the physical and social realities of users’ daily lives [17]. This disconnect often results in technologies that fail to meet users’ actual needs, leading to limited effectiveness and high rates of abandonment [15]. These shortcomings highlight the importance of grounding technological innovation in the lived experiences of those concerned. The principle of “nothing about us without us” should guide every stage of design, development, and implementation when technologies are intended for people with disabilities [17]. In addition to user involvement, contextual factors such as environmental, social, and cultural conditions strongly influence usability and adoption [6]. Variability in lighting, acoustic conditions, spatial organization, and social norms around receiving assistance can significantly shape navigation experiences [6]. These context-dependent factors justify the need for a flexible, adaptive, and holistic user-centered design (UCD) approach that accounts for diverse real-world conditions [6]. Importantly, this study represents the first multiphase, interdisciplinary co-design process for indoor navigation technology for people with visual impairment conducted across Canadian rehabilitation centers. By situating the research within Canada’s technological and clinical landscape, we demonstrate its novelty and contribution beyond reiterating the importance of co-design. This work provides a replicable framework that integrates participatory design, inclusive technology development, and cross-sector collaboration to advance the field of assistive navigation.

### Limitations of Existing Navigation-Based AT

Despite technological progress, many NATs remain underused due to poor alignment with users’ lived contexts [17], leading to high abandonment rates. Solutions often fail because they do not adequately consider dynamic indoor environments, usability simplicity, or the importance of multisensory cues. Moreover, trust is quickly eroded when devices produce errors or require complex interactions, reinforcing the principle “nothing about us without us” as essential for relevance, usability, and acceptability. To reduce the frequent nonadoption and abandonment of NATs, three main approaches have recently gained prominence in the development process: UCD, participatory co-design, and transdisciplinary collaboration [17].

### UCD Approach

The UCD approach in the development process of AT has grown in recent years [17]. UCD, and more specifically inclusive design, is an approach aimed at developing interfaces, artifacts, products, and services that are usable, appropriate, and accessible to the widest possible range of users, while respecting the constraints of the design specifications [18]. UCD is a method for creating usable products and systems, based on a set of techniques, processes, and methods that all place the user at the center of each stage [17]. Chavarria et al [19] highlight the importance of human-centered design (HCD) in AT development. UCD plays a key role in the development of ATs, as it prioritizes users’ needs and contexts through continuous engagement. For instance, Tang et al [20] underscore the role of wearable multimodal systems as a key component of inclusive design strategies. This iterative approach ensures that ATs are practical, accessible, and effective [21]. Improving the implementation of UCD in AT development and strengthening the evidence base regarding its implementation and outcomes are crucial [17]. The ISO (International Organization for Standardization) 9241-210:2019 standard provides internationally recognized principles for UCD [22]. To better engage with the realities of people with visual impairment, it is important to enhance the capacity of development teams in UCD, its principles, and components, and to improve the planning of UCD implementation [17]. UCD is guided by six key principles: (1) an explicit understanding of users, their tasks, and environments; (2) continuous user involvement throughout the design and development process; (3) design decisions informed and refined by user-centered evaluation; (4) an iterative development process; (5) consideration of the overall user experience; and (6) a multidisciplinary design team bringing diverse skills and perspectives [17].

### Participatory Co-Design Approach

Co-design is a meaningful engagement of the end user at all stages of the process [18]. Participatory co-design approaches are crucial for a thorough understanding and engagement of end users, ensuring more appropriate and user-friendly products and services [19]. Gathering user information and feedback at each stage of the design and development process is essential, involving all stakeholders: users, designers, and other relevant parties [21]. A participatory co-design team comprises a range of multidisciplinary skills and viewpoints [19]. The significance of individual contributions, as highlighted in the participatory design framework by Sanders [23], lies in the valuable input each stakeholder provides at every phase of the design process. These key players are vital sources of creativity and inspiration, generating relevant and innovative ideas. Considering the diversity of the target population should not be an afterthought, but rather a fundamental prerequisite for any design, integrated from the outset in the needs specifications [21]. This approach recognizes each user as a unique individual with their own abilities, experiences, and characteristics [21]. It considers the capabilities and limitations of all potential users. Technologies designed in this way will be usable by the widest possible range of individuals, across a variety of situations, maximizing reach and impact [21]. Beyond conventional participatory approaches, sociomaterial perspectives show that facilitators and constraints in co-design emerge from dynamic configurations between actors, artifacts, material environments, and organizational routines [24]. Rong and Hansopaheluwakan-Edward [24] demonstrate that in co-design projects involving people with visual impairments, certain constraints such as material properties, organizational rules, or interaction modalities can also act as facilitators depending on how they are configured. This sociomaterial perspective informs our own co-design mechanisms, including the selection of design artifacts, the structuring of workshop activities, and the governance model across interdisciplinary teams. It also justifies key methodological trade-offs implemented to ensure effective participation and sustained alignment between technical, clinical, and experiential considerations [24].

### Transdisciplinary Approach

Transdisciplinarity opens the possibility for cross-fertilization among different fields of knowledge and collaboration with people with visual impairment as potential end users and with their stakeholders [17]. Cross-fertilization across engineering, social, and clinical sciences is necessary [17]. A multidisciplinary approach, integrating diverse expertise, ensures that user needs are met [17]. Considering universal design is of great significance as it aims to optimize product design for maximum accessibility and to ensure that mainstream design is accessible to everyone [17]. The goal is for this approach to provide outstanding solutions that function equally well for users with disabilities as for those with higher abilities [22]. Adherence to the principles of nonstigmatization and nondiscrimination is essential because users are unlikely to adopt, appreciate, or purchase products that stigmatize them or emphasize their disability; however, they possess the capability and expertise to suggest ways to mitigate such stigmatizing effects [21]. Multidisciplinary teams would not only address technology-related issues but also those pertaining to user functionality. Although AI has made significant advancements in indoor navigation for people with visual impairments, its integration into an intersectional and participatory approach remains largely unexplored. These approaches are often fragmented, lacking systematic integration of people with visual impairment’s lived experiences and cross-disciplinary expertise, which reduces the impact and sustainability of innovations [25]. This study aims to address this gap by considering the diverse experiences and lived realities of users while bringing together a broad range of expertise, including researchers, rehabilitation professionals, and industry stakeholders, to ensure a truly inclusive technology that meets varied needs. Hence, the interest in conducting this study.

### Research Goal and Objectives

Our research aims to develop an inclusive and user-centered indoor navigation platform that effectively addresses the needs of people with visual impairment in Canada. Specifically, it is about (1) conducting qualitative research to understand the specific navigation challenges faced by people with visual impairment in various indoor environments; (2) implementing a UCD approach that ensures continuous user involvement at all stages of the platform’s development, allowing for iterative feedback and refinement; (3) engaging stakeholders, including users, designers, and academics, in a collaborative workshop to gather insights and ideas that drive the design process, ensuring that the platform is user-friendly and accessible; (4) fostering cross-disciplinary partnerships among a range of fields (engineering, social sciences, and clinical sciences) to ensure the design process incorporates diverse perspectives and expertise; (5) testing and evaluating the platform in controlled and real-world environments with real users, collecting feedback to assess usability, functionality, and overall user experience; and (6) sharing the research outcomes and best practices for developing NATs with the broader community, including industry partners and policymakers, to promote wider implementation. By achieving these objectives, the research aims to deliver a robust indoor navigation solution that enhances the independence and quality of life of people with visual impairment.

## Methods

### Overview

The Edge A-Eye project used a participatory co-design methodology in which people with visual impairment, specialists in vision rehabilitation, engineers, human-centered computing experts, AI, computer science researchers, as well as community partners collaborated throughout all stages of development. This approach ensured that experiential knowledge, scientific evidence, and technological expertise were fully integrated into an inclusive and ethically grounded innovation process, consistent with the mechanisms of mutual learning and shared decision-making described in participatory co-design literature [26]. The sustainability of digital health innovations depends on the continuous engagement of stakeholders, the active involvement of users at every stage, and stable collaborative governance [27]. The participatory structure prioritized transparency, responsibility, interdisciplinarity, and direct responsiveness to user-identified needs, in alignment with best practices in equitable, human-centered, and interdisciplinary design processes [28].

### Ethical Considerations

Our study adheres to the ten guiding principles outlined in the Montreal Declaration on the Ethics of AI, namely: well-being, respect for autonomy, protection of privacy and personal data, solidarity, democratic participation, equity, inclusion of diversity, caution, responsibility, and sustainable development [29]. We adhered to ethical principles, in accordance with the Declaration of Helsinki [30], including approvals from the CERC (Clinical Research Ethics Committee) of the University of Montreal (CERC-2022‐1800; January 13, 2023), CERC-2024‐2051 (May 3, 2024), and the Centre de Recherche Interdisciplinaire en Réadaptation du Montréal Métropolitain (CRIR MP-50-2024-51; May 3, 2024). Participants provided free, voluntary, and informed consent; anonymity and data confidentiality were ensured. Source documents were accessible via VoiceOver (ResonanceVista) on adapted computers or smartphones. All data collected were anonymized and documented in a Microsoft Excel (Microsoft Corp) file coded for deidentification, while signed consent forms were securely stored in a password-protected folder, thereby guaranteeing confidentiality and adherence to ethical requirements. These measures are essential to ensure the integrity of the research and the respect for ethical values [31]. Participants received compensation of CAD $15 per hour (a currency exchange rate of CAD $1=US $0.79 was applicable).

### Research Team Composition and Participation Structure

The Edge A-Eye project was spearheaded by a multidisciplinary consortium bringing together four academic institutions and several industry partners: the School of Optometry and the School of Public Health at Université de Montréal, École de Technologie Supérieure (ÉTS), in collaboration with Mathematics of Information Technology and Complex System, IVADO (Institut de Valorisation des Données), and Broadcom. This alliance integrated expertise in vision science, public health, AI, human‑centered computing, computer science, and community engagement, ensuring both methodological rigor and technical relevance throughout all stages of development [26,32]. To translate participatory design principles into practice, we developed a participation matrix mapping the roles and contributions of each stakeholder group (people with visual impairment, vision rehabilitation specialists, researchers, and industry partners) across the eight co-design steps (Table 1). This structured approach demonstrates that people with visual impairment were not passive informants but active co-designers, contributing experiential knowledge to feature prioritization, prototype testing, and iterative refinements. Rehabilitation specialists ensured clinical integration, while industry partners provided insights on technical feasibility and scalability. Researchers coordinated activities, managed data analysis, and upheld ethical standards [32,33]. The leadership team comprised a professor of vision science (JPN), a professor of public health (HA), an industry expert (BP), and a professor of technological engineering (KKN). They were supported by five vision science researchers (SSK, PRP, DA, SMR, and NG-R) and three engineering researchers (PAN, JG, and HN), who contributed to participant recruitment, study coordination, and prototype development. Weekly coordination meetings fostered collaborative and iterative decision-making, reinforcing the participatory nature of the current project [34]. Partnerships with community organizations and rehabilitation centers, including the CRIR, CNIB (Canadian National Institute for the Blind) Foundation, ASAMM (Association des Sports Pour Aveugles du Montréal Métropolitain), Institut Nazareth et Louis-Braille (INLB), Lethbridge-Layton-Mackay Rehabilitation Center (LLMRC), and RAAMM (Regroupement des Aveugles et Amblyopes du Montréal Métropolitain), ensured the integration of lived experiences and promoted equity and inclusion. This framework guaranteed that empowerment was not symbolic but embedded in the design process, fully aligned with the principle of “nothing about us without us” [32,33]. Importantly, people with visual impairment were not only consulted as informants but actively engaged as co-designers of the Edge A-Eye platform, in line with established participatory design principles emphasizing end users’ involvement and empowerment [32,33]. This continuity enriched the depth and consistency of experiential insights, reduced variability, and fostered trust among stakeholders. It also enabled cumulative learning, where feedback from earlier stages informed subsequent design decisions, thereby strengthening ecological validity and usability of the Edge A-Eye platform [18,21]. Implementing a participatory co-design methodology within a large interdisciplinary consortium required active coordination to manage expectations and ensure effective communication across diverse teams. Differences in disciplinary vocabularies and priorities between technical, clinical, and user partners occasionally led to misalignment regarding feasibility and development timelines. These challenges were addressed through structured coordination mechanisms, including regular interdisciplinary meetings and iterative validation checkpoints, which facilitated clarification of terminology, alignment of objectives, and transparent decision-making throughout the development process. Table 1 presents the participation matrix.

**Table 1.** Participation matrix.

Co-design stage	People with visual impairment	Rehabilitation specialists	Researchers	Industry partners
Needs assessment (focus groups and interviews)	Shared lived experiences; identified barriers and facilitators	Validated clinical relevance	Facilitated sessions; analyzed data	Not available
Prioritization of features (workshop)	Ranked priorities; proposed functionalities	Linked priorities to rehabilitation practices	Synthesized themes; documented decisions	Provided feasibility insights
Preliminary design	Reviewed wireframes; suggested accessibility improvements	Ensured ergonomic and clinical integration	Developed prototypes	Assessed technical constraints
Iterative development	Tested prototypes; provided usability feedback	Recommended adjustments for safety	Implemented refinements	Optimized performance
Controlled testing	Evaluated navigation tasks in laboratory settings	Observed and advised on mobility strategies	Collected and analyzed data	Supported technical troubleshooting
Real-world testing	Simulated navigation in authentic environments	Monitored functional outcomes	Coordinated logistics; analyzed results	Assisted with deployment
Dissemination of results	Shared experiential insights at events	Advocated for clinical adoption	Presented findings at conferences	Explored commercialization pathways
Ongoing evaluation	Continuous feedback for improvements	Ensured alignment with rehabilitation standards	Maintained iterative updates	Planned scalability

### Study Design

This study (May 15, 2024, to December 5, 2025) adopted a participatory, intersectoral co-design approach to develop a NAT tailored to the needs of people with visual impairment in Canada. People with visual impairment were actively engaged alongside vision rehabilitation specialists, academics, and industry stakeholders to ensure that the technology was both functional and responsive to real-world challenges. The current project followed the principles of transdisciplinary AT development [35] and UCD [17], which emphasize users’ needs, motivations, and behaviors throughout the design process [36]. To minimize bias, no specific technology was introduced during the current study, allowing participants to consider a range of solutions, including human assistance or physical accessibility measures. The reference framework used is Bird et al [37] because it proposes a generative co-design approach centered on users, which is essential for integrating the real needs of people with visual impairment into technological development. It is structured into three phases: predesign (mobilization and understanding of needs), co-design (collaborative creation and uncovering of latent needs), and postdesign (validation and recommendations). This model promotes the acceptability and adoption of health innovations through the active participation of stakeholders [37]. The current study’s methodology and findings were thoroughly documented in accordance with the COREQ (Consolidated Criteria for Reporting Qualitative Research) checklist [38], presented in Checklist 1. This framework ensures comprehensive reporting of essential elements, including details about the research team, study design and procedures, setting, results, data analysis, and interpretation [38]. Structured into eight steps outlined below, the process ensured that the perspectives of people with visual impairment remained central throughout, while promoting strong collaboration and sustained engagement among all stakeholders [33,36].

### Integrated Scientific Reference Frameworks

**Figure 1.**
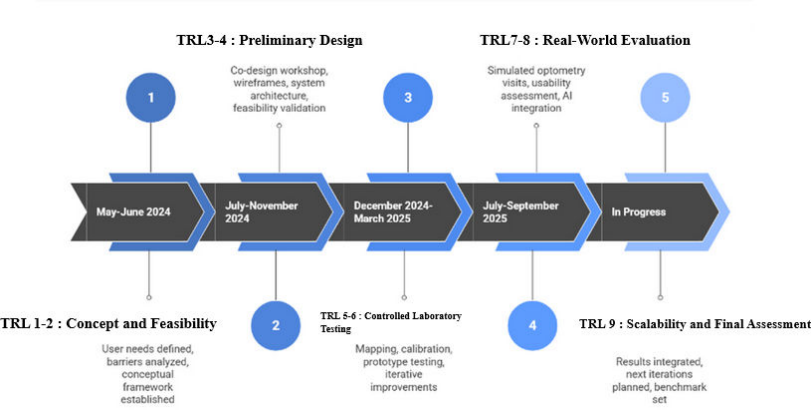
Ongoing monitoring and evaluation of the Edge A-Eye project. :

**Figure 2.**
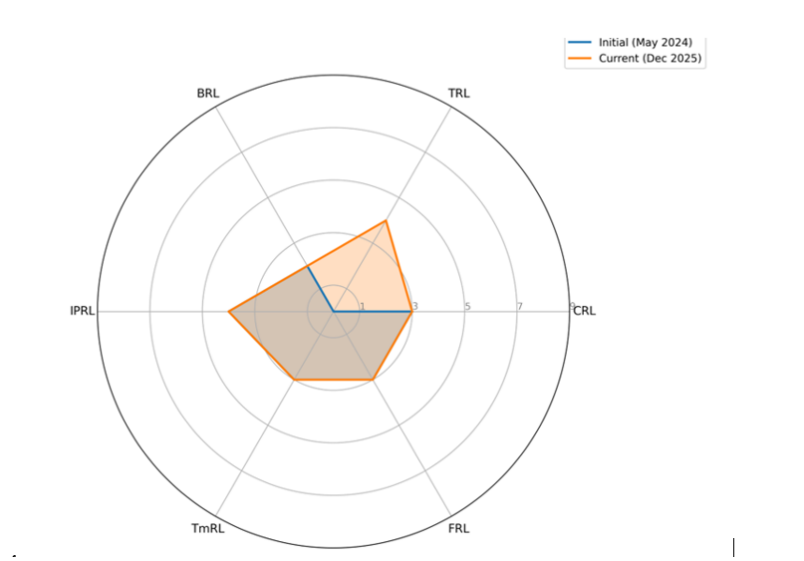
TRL progression across 55 axes, comparing the initial state (May 2024) versus the current state (December 2025). BRL: Business Readiness Level ; CRL: Commercial Readiness Level ; FRL: Financial Readiness Level ; IPRL: Intellectual Property Readiness Level :TMRL: Team/Management Readiness Level ;TRL: *Technology Readiness Level.*

The Edge A-Eye project is built on three complementary methodological pillars to ensure inclusive and sustainable innovation:

1. Technology acceptance model (Davis [39]): this model was applied to examine factors influencing technology adoption by identifying both barriers and facilitators. This approach enabled a clear understanding of actual user needs and their translation into design requirements for an adapted indoor navigation system, thereby enhancing acceptability and reducing the risk of abandonment. The model emphasizes two key determinants of technology acceptance: perceived usefulness and ease of use, which strongly shape the integration of ATs into everyday life.
2. Generative co-design framework (Bird et al [37]): this framework structured the participatory and cross-sector process, ensuring continuous involvement of end users and stakeholders. It guarantees that design decisions reflect real needs and lived contexts through prototypes tested in both laboratory and real-world conditions.
3. Technology readiness level (TRL; KTH Innovation [40]): the TRL framework was explicitly used as a development governance tool to structure decision-making, progression criteria, and validation milestones throughout the Edge A-Eye project. Rather than serving solely as a post hoc indicator of technological maturity, the TRL framework was integrated prospectively to guide the sequencing of design, testing, and evaluation activities. Each transition between TRL stages was informed by predefined feasibility criteria, user feedback, and multidisciplinary consensus involving researchers, clinicians, industry partners, and people with visual impairment. This governance approach ensured that development decisions were grounded in empirical evidence, usability outcomes, and contextual feasibility, while preventing premature claims of maturity or scalability. The TRL framework thus functioned as a structuring and regulatory mechanism supporting iterative refinement, risk management, and transparency across this project’s lifecycle. The operationalization of this governance framework and its application across successive TRL stages are detailed in the ongoing monitoring and evaluation section and summarized visually in Figures 1 and 2.

In summary, combining the technology acceptance model for acceptability, Bird for co-design, and TRL for maturity creates an integrated logic chain that strengthens the relevance, robustness, and sustainability of the innovation.

### Steps of the Co-Design Process

**Figure 3.**
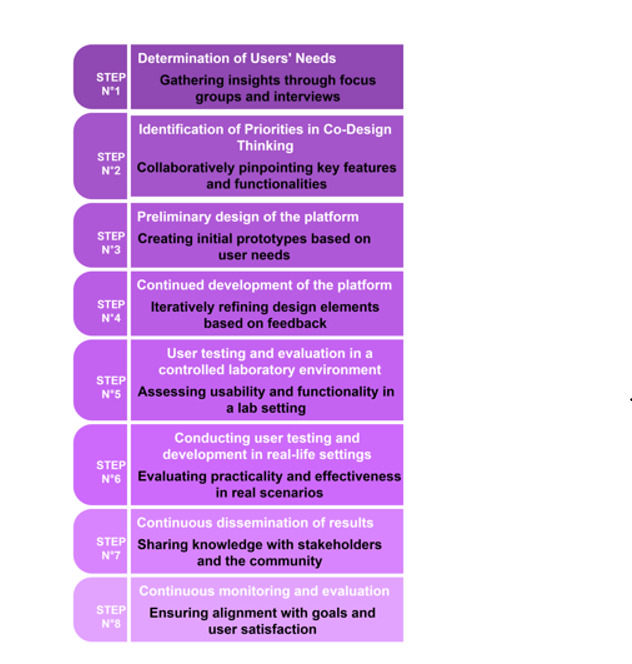
Structured steps of the co-design process.

This study resulted in the development of eight structured co-design steps, as shown in Figure 3. The structured methodology, aligned with scientific rigor and ethical standards, yielded these key steps. First, regarding the determination of users’ needs, focus groups and interviews provided essential qualitative insights into the lived experiences, mobility challenges, and expectations of people with visual impairment. This foundational step ensured that the platform was grounded in real user contexts [36]. Second, regarding the identification of co-design priorities, stakeholders collaboratively identified key features, navigation challenges, and accessibility requirements. This prioritization ensured alignment with user preferences and everyday mobility needs [35,37]. Third, regarding the preliminary platform design, initial prototypes were developed based on users’ expressed needs. Early conceptualization enabled rapid feedback and ensured that proposed solutions matched user expectations [36]. Fourth, regarding the iterative platform development, ongoing user feedback guided continuous refinement of functionalities, interface elements, and navigation cues. This iterative process reinforced the adaptability and responsiveness of the design [37]. Fifth, regarding the controlled-environment testing, user evaluations conducted in a laboratory environment yielded important insights into usability and interface functionality, supporting improvements before real-world testing [35]. Sixth, regarding the real-world testing, field testing confirmed the practical relevance, accessibility, and feasibility of the platform in authentic indoor environments. Real usage scenarios provided rich data on actual navigation behaviors and enhanced ecological validity [25,35]. Seventh, regarding the continuous dissemination of results, regular sharing of findings with partners, stakeholders, and community organizations strengthened collaboration and supported transparency throughout the development process [41]. Eighth, regarding the ongoing monitoring and evaluation, continuous assessment ensured alignment with user expectations and project objectives. Iterative adjustments maintained platform relevance and enhanced long-term user satisfaction [37].

37

To make sprint decisions traceable, user-reported feedback was prioritized using a three-factor rubric: (1) frequency (number of participants or scenarios affected across sessions), (2) severity (risk to safety or task completion; high potential collision, misrouting, or loss of situational awareness), and (3) usability impact (cognitive load, clarity of cues, and effort required to operate hands-free). Items with high severity were addressed first, even if low in frequency; ties were resolved by usability impact. Each sprint started with a prioritization board (backlog review) and ended with a verification step (targeted retesting of high-priority fixes in the laboratory, then real-world scenarios). Representative examples include refining turn-by-turn prompts at decision points to lower cognitive load (medium severity or high impact).

### Application of ISO 9241-210:2019 Principles

The co-design methodology adopted in this study was explicitly aligned with the six principles outlined in ISO 9241-210:2019, which provides internationally recognized guidance for HCD of interactive systems [22]. These principles include (1) an explicit understanding of users, tasks, and environments; (2) active user involvement throughout design and development; (3) iterative design processes; (4) design driven and refined by user-centered evaluation; (5) consideration of the entire user experience; and (6) a multidisciplinary design team. In practice, these principles were operationalized through continuous engagement of people with visual impairment and visual rehabilitation specialists across all eight co-design stages, iterative prototype testing in both controlled and real-world settings, and integration of expertise from engineering, vision science, and human-centered computing. This alignment ensured that the Edge A-Eye platform was developed in accordance with global standards for usability, accessibility, and inclusivity.

### Participant Recruitment and Eligibility

A purposive sampling strategy was used to recruit both people with visual impairment and vision rehabilitation specialists. This approach aligns with qualitative research standards emphasizing the involvement of key informants whose experiential knowledge is essential for co-design processes [42]. Vision rehabilitation specialists were selected for semistructured interviews conducted in French, with inclusion criteria requiring that participants be practicing rehabilitation specialists residing in Montréal. Recruitment of people with visual impairment followed three complementary components: (1) French- and English-language focus groups, simulating indoor navigation in grocery store and clinic scenarios to identify barriers and facilitators; (2) a co-design workshop at IVADO, where users collaboratively prioritized needs and defined core functionalities of the navigation platform; and (3) real-life scenario simulations at the LLMRC, evaluating feasibility, acceptability, and user experience in authentic environments. Information sheets and electronic consent forms were distributed via email through partner organizations (CRIR, CNIB Foundation, ASAMM, INLB, and RAAMM) and posted on social media. Inclusion criteria for people with visual impairment required being aged ≥18 years, residence in Canada, self-identification as visually impaired (including low vision, blindness, and deafblindness), semi-independent travelers, regular smartphone use for at least one year, ability to communicate in French or English, basic internet and Zoom (Zoom Communications, Inc) proficiency, stable internet access, absence of intellectual or cognitive disabilities, and voluntary written consent to participate. To ensure diversity and representativeness, the sample considered variations in age, gender, ethnic and cultural background, and type of vision loss. Individuals who did not meet the eligibility criteria were excluded. Participants who were determined eligible by the research team were contacted about their interest in participating in additional research by the laboratory. They were selected at each stage to prioritize a diversity of profiles. An estimated sample size of 13 participants was determined for each stage, based on similar qualitative studies conducted in this field. However, data collection continued until saturation was reached. Participant continuity was ensured across the different phases. A core group of 5 participants (4 people with visual impairment and 1 specialist for people with visual impairment or vision-rehabilitation) was maintained throughout the process, which allowed for improved iterative refinement and ensured coherence in design decisions.

### Data Collection

The data collection strategy combined multiple instruments to ensure a comprehensive Edge A-Eye platform during the co-design process. Together, all tools provided a structured framework that covered the entire co-design cycle. By integrating qualitative approaches, the methodology enabled a robust triangulation of findings, thereby strengthening the validity and reliability of the study outcomes.

### Focus Group Sessions

To provide initial insights into the needs of people with visual impairment in our current Edge A-Eye project (to inform this study), 2 web-based focus groups were conducted between February and June 2023 to explore barriers and facilitators related to indoor navigation for people with visual impairments. One session was held in English and the other in French via Zoom, each lasting approximately 2 hours. Moderators followed a pretested guide to ensure consistency and encouraged participants to share experiences or imagine completing tasks in 5 everyday scenarios: coffee shop, clinical visit, big-box store, bus travel, and party.

Sessions were recorded, transcribed (English automated and French manual), and validated. Themes were coded independently, with discrepancies resolved by JPN. A total of 2 weeks later, participants ranked the identified themes by importance via email, enabling prioritization of barriers and facilitators for each scenario. The results that informed the initial stages of the Edge A-Eye project were recently published by the laboratory [43].

### Semistructured Interviews

On July 24, 2024, a total of 2 vision rehabilitation specialists with visual impairments participated in semistructured interviews conducted via the Zoom platform. One of them joined the research team at a later date to collaborate closely throughout all stages of the Edge A-Eye project. Each session lasted approximately 2 and a half hours and allowed participants to express their perspectives in either language while discussing simulations of 2 scenarios: navigating in a grocery store and attending a clinic appointment. Their expertise provided valuable insights into the barriers and facilitating factors associated with the use of ATs for indoor navigation in Canada, thereby contributing to a deeper understanding of the real needs of people with visual impairment. The interviews were facilitated by SSK, who ensured the structured flow of the interview, managed time effectively, and took detailed notes. A pretested semistructured interview guide (Multimedia Appendix 1) was used to support consistency and rigor in data collection.

### Co-Design Workshop

The co-design workshop was hosted on November 1, 2024, by IVADO in Montreal, Quebec. Building on insights gathered from interviews and focus groups, the research team designed and pretested a semistructured interview guide (Multimedia Appendix 2) to frame and support the co-design thinking process. The primary objective of this workshop was to prioritize the needs identified by people with visual impairment, ensuring that their experiential knowledge guided the development of the Edge A-Eye platform from the outset. The workshop brought together 22 participants, including 4 people with visual impairment of diverse ages, genders, and conditions, 1 vision rehabilitation specialist, 4 vision science researchers, 11 technology design researchers, 1 representative from the IVADO, and 1 industry stakeholder. All stakeholders facilitated constructive discussions. A structured brainstorming and prioritization process was used to identify the key features and functionalities most relevant to people with visual impairment. Notetakers were assigned to each table, which included at least 1 visually impaired participant, 1 industry representative, and 1 academic researcher. Each team generated lists of priorities in separate Microsoft Excel (Microsoft Corp) files, and designated presenters reported thematic categories to SSK. These themes were compiled and reviewed for accuracy by team members. The responses were then synthesized and summarized collaboratively by SSK, JPN, DA, PRP, and PAN, and subsequently used to produce presentations and documentation aimed at effective knowledge transfer.

### Simulating Scenarios in Real-Life Contexts

**Figure 4.**
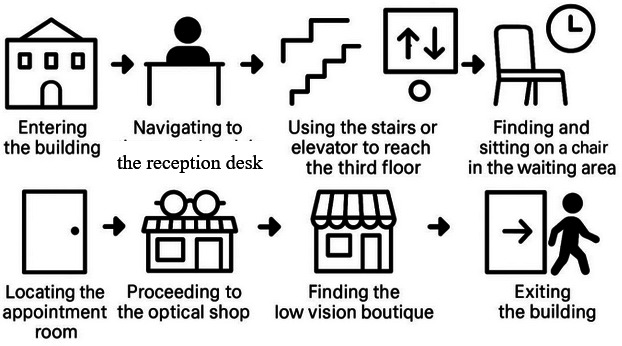
Sequential tasks for real-life clinical testing for Edge A-Eye.

Real-life testing of people with visual impairment with vision rehabilitation specialists and vision researchers was conducted at the LLMRC site between July 17 and September 19, 2025. For this evaluation, a simulated optometry visit was designed, consisting of eight sequential tasks: (1) entering the building, (2) navigating to the reception desk, (3) using the stairs or elevator to reach the third floor, (4) finding and sitting on a chair in the waiting area, (5) locating the appointment room, (6) proceeding to the optical shop, (7) finding the low-vision boutique, and (8) exiting the building. Figure 4 presents the sequential tasks of the simulated optometry visit.

As part of this real-life indoor navigation simulation stage, four complementary data collection instruments were used to enable a nuanced and multidimensional understanding of the needs and experiences of people with visual impairment:

1. Eligibility questionnaire (Multimedia Appendix 3): administered during the initial recruitment phase, before any testing. It consisted of closed-ended questions designed to verify inclusion and exclusion criteria, as well as to collect sociodemographic data (age, gender, visual condition, and experience with ATs). Its early administration ensured that only eligible participants were included in this study, while also establishing a baseline profile of the sample.
2. Prescenario questionnaire (Multimedia Appendix 4): administered immediately before the navigation scenario. It included questions aimed at assessing participants’ expectations, confidence levels, and comfort regarding indoor navigation with ATs. By being administered just before the scenario, it captured initial perceptions and provided a reference point for comparison with postscenario feedback.
3. Walking interview questions (Multimedia Appendix 5): conducted interactively during the real-life scenario. This semistructured guide comprises open-ended questions designed to capture real-time perceptions, obstacles encountered, and immediate reactions related to indoor navigation. Notetakers documented responses during navigation, ensuring the collection of contextualized qualitative data. The objective was to complement quantitative measures with rich insights into user experience, frustrations, and facilitators.
4. Postscenario questionnaire (Multimedia Appendix 6): administered immediately after the completion of the navigation scenario. It included both closed-ended and open-ended questions, aimed at evaluating satisfaction, perceived effectiveness of the technology, usability, and suggestions for improvement. Administered while impressions were still fresh, it allowed the documentation of real navigation needs as well as recommendations for refinement.

### Data Analysis

Thematic analysis, conducted through an inductive-deductive approach [44], identified user needs, preferences, and concerns regarding accessibility, safety, and privacy [43]. The inductive approach allowed themes to emerge directly from the data, while the deductive approach ensured consistency with existing theoretical frameworks on AT and user experience [44]. The combination of these perspectives provided a robust analytical framework, blending structured reasoning with open-ended exploration, and ensuring a comprehensive understanding of qualitative data [44]. The thematic analysis procedure followed the Braun and Clarke [45] model with NVivo (Lumivero). Interviews and focus groups were fully transcribed and validated for accuracy. The detailed procedure of this thematic analysis, applied to focus groups and expert interviews, is described in a recently published study by our laboratory [43]. In parallel, a descriptive qualitative approach was adopted to synthesize feedback and identify recurring ideas related to the needs of people with visual impairment and their co-design experiences. Meeting transcripts, facilitator logs, and session summaries were reviewed by the research team to highlight recurring themes, key insights, and decisions made collaboratively with participants. Further, 2 researchers (JPN and HA) independently analyzed the session notes to ensure consistency of interpretation and strengthen analytic trustworthiness through iterative discussion and consensus. A detailed log of all interactions related to the Edge A-Eye project was maintained, and information was systematically organized into categories to support the decision-making process. Following the evaluation of these sessions, as well as qualitative interviews, the priority areas necessary for the co-design of the application were defined. Table 2 presents the six phases of thematic analysis as described by Braun and Clarke [45], outlining the progression from familiarization with the data to the final reporting of results.

**Table 2.** Six phases of thematic analysis.

Steps	Description	Team members involved
Familiarization	Reading and rereading full interview transcripts and written statements to become deeply familiar with the data and identify initial ideas.	SSK, DA, PRP, HA, JPN
Generating initial codes	Organizing all data into meaningful groups through the creation of preliminary codes.	SSK, DA, PRP
Searching for themes	Grouping codes from transcripts and Excel files into subthemes and then into overarching themes.	SSK, DA, PRP, JPN, HA
Reviewing themes	Checking the coherence of themes in relation to the codes and the entire dataset, resolving discrepancies through discussion.	SSK, DA, PRP, JPN, HA
Defining and naming themes	Clearly defining the scope, content, and labels of each theme.	SSK, DA, PRP, HA, JPN
Producing the report	Synthesizing analyses and producing the final report of results.	SSK, DA, PRP, HA, JPN

### Preliminary Analyses to the Edge A-Eye Team

To accelerate the transmission of preliminary results among members of the Edge A-Eye research team and promote an agile framework for technology development, descriptive statistics were shared on a weekly basis during testing. This was based on the results from participants in each of the four phases of real-life testing, indicated above. This included sociodemographic information, the visual status of participants, the ability of participants to complete tasks, the use of technological devices, as well as distance and direction errors of participants. Although more detailed analyses are underway for the scenarios in real-life contexts, this is beyond the scope of this manuscript, illustrating the co-design methodology carried out during the Edge A-Eye project.

## Results

### Overview

This section summarizes the main findings from the co-design process and subsequent evaluations. It begins by outlining the participants’ characteristics to highlight the diversity of profiles included in this study, followed by a description of the key stages in the development of the Edge A-Eye platform, the identified needs, prioritized requirements, and performance outcomes observed during real-world testing.

### Evolution of the Edge A-Eye System Across Co-Design Phases

The steps of the co-design process are shown in the results below, as well as how each of these steps informed the subsequent steps of design and development of the platform.

### Determining User Needs (Focus Groups and Semistructured Interviews)

The needs assessment phase combined qualitative insights from two web-based focus groups and semistructured interviews with adults living with visual impairments across Canada to inform the subsequent phases of this project. Across all datasets, the analysis converges toward a coherent understanding of the factors shaping indoor navigation for people with visual impairment, emphasizing the interaction between environmental accessibility, human support, and the limitations of existing technologies. A total of 20 adults with visual impairments participated in the two focus groups (one in English and one in French). Participants represented a wide diversity of demographic and clinical profiles, including age groups (18‐49 and 50+ years), levels of vision (low-vision, legal blindness, or complete blindness), etiologies of vision loss (visual acuity loss, visual field loss, or combined deficits), and mobility aids (white cane, guide dog, support cane, walker, or none). All were regular smartphone users, fluent in English or French, and able to travel independently at least occasionally. This purposeful sampling ensured maximum variability across navigation habits and accessibility needs. Data were collected between February and June 2023, beginning with the two focus groups, followed by an individual email-based ranking exercise to consolidate and prioritize user-reported needs. All participants completed each component of this study. In TA cross-scenarios, participants consistently reported structural barriers that undermine autonomy during indoor navigation. These include inaccessible or inconsistent signage, cluttered or unpredictable spaces, poor lighting or contrast, difficulties in locating specific destinations, and ineffective or uncomfortable social interactions when requesting help. Conversely, users emphasized that supportive staff, advance preparation, reliable multisensory cues, and access to clear and accessible information significantly facilitate safe and confident mobility. These facilitators were echoed across both language groups and multiple contexts, confirming their central role in indoor wayfinding. The need for coherent environmental design, context-aware guidance, and multimodal orientation cues also emerged strongly from the focus groups. Participants described scenarios where auditory cues, tactile markers, or consistent spatial organization substantially improved their navigation performance. At the same time, concerns were raised regarding the usability and reliability of current technologies, particularly in complex indoor spaces where GPS is not effective and where mobile apps may provide insufficient precision or unclear instructions.

To complement user perspectives, 2 vision rehabilitation specialists participated in this study as expert validators. Their contribution was essential for linking experiential needs to clinical practice. They confirmed the centrality of personalized and contextually integrated solutions, highlighting that indoor navigation support must be adaptable to diverse functional profiles and embedded into broader rehabilitation pathways. Their recommendations reinforced the importance of developing systems capable of offering fine-grained directional guidance, dynamic distance cues, reliable landmark recognition, and flexible notification settings adapted to users’ mobility strategies. Overall, the combined findings from the focus groups, semistructured interviews, and expert validation identify a clear set of priorities for accessible indoor navigation: (1) ensuring cohesive and perceivable environmental information, (2) integrating multisensory cues that align with real-world mobility practices, (3) designing intuitive and trustworthy technologies, and (4) strengthening inclusive human support systems that promote autonomy without compromising user comfort. These results directly informed the subsequent co-design and prototype development phases, ensuring that Edge A-Eye remains grounded in the lived realities and expectations of its intended users. The barriers and facilitators by scenario are summarized in Table 3.

**Table 3.** Barriers and facilitators (focus groups French or English).

Scenario	Facilitators (English)	Facilitators (French)	Barriers (English)	Barriers (French)
Coffee shop	Human assistance; preparation; accessible physical space	Human assistance; accessible payment; sensory cues	Difficulty locating exact spot; poor interactions; inaccessible signage	Lighting; inaccessible signage; mobility difficulties
Hospital or clinic	Human assistance; mobility support; preparation	Human assistance; preparation; accessible signage	Locating exact destination; poor interactions; mobility difficulties	Inaccessible signage; unsuccessful interactions; mobility difficulties
Big box store	Human assistance; preparation; accessible website	Accessible website; preparation; human assistance	Mobility difficulties; locating exact place; locating specific item	Asking staff; inaccessible signage; mobility difficulties
Party	Preparation; accessible space; human assistance	Human assistance; accessible space; alternative strategies	Mobility challenges; poor interactions; loud environment	Fear of spilling; noise; mobility difficulties
Bus	Accessible signage; preparation; accessible space	Accessible signage; accessible space; human assistance	Inaccessible signage; poor interactions; mobility difficulties	Inaccessible signage; locating exact place; unexpected events

### Prioritization of Requirements (Co-Design Workshop)

The intersectoral co-design workshop held on November 1, 2024, brought together people with visual impairment, a vision rehabilitation specialist*,* researchers from the University of Montreal or ÉTS, representatives from IVADO or Mathematics of Information Technology and Complex Systems, and contributors from the INLB. This collaborative session played a pivotal role in translating the needs expressed during focus groups, walking interviews, and real-world evaluations into a structured set of actionable design requirements. The outcomes were organized around three core axes: physical accessibility, virtual accessibility, and privacy or security.

### Physical Accessibility

This axis captures elements related to the user’s interaction with the built environment. Discussions highlighted the need to support safe, efficient navigation in complex indoor settings, consistent with the barriers identified during earlier stages (eg, inaccessible signage, low contrast, difficulty identifying doors, and risk of collisions). Priority requirements were (1) door-level guidance, including precise identification and approach support; (2) accessible emergency evacuation plans; (3) automated sign reading and audio announcement using camera-to-speech conversion; (4) AI-based object recognition to support proactive awareness; (5) emergency mode for urgent navigation needs; (6) indoor maps translated into audio instructions; (7) obstacle detection with real-time feedback; and (8) noise adaptive alerts and haptic notifications.

These priorities reflect the need for robust environmental interpretation features that enhance independence and reduce cognitive strain.

### Virtual Accessibility

Virtual accessibility encompasses the interaction between the user and the digital interface, including personalization, control mechanisms, and flexibility in information delivery. Participants emphasized simplicity, adaptability, and the ability to tailor navigation to situational needs. Priority requirements were (1) voice or haptic command options enabling hands-free use; (2) fully customizable settings (volume, speech rate, contrast, or language); (3) flexible distance representations (meters, steps, or clock-face orientation); (4) exhaustive but filterable information, preventing overload; (5) two navigation modes: focused mode for essential, task-specific guidance and exploratory mode for broader environmental awareness; (6) estimated time of arrival or floor indicators for vertical navigation; (7) full compatibility with screen readers (eg, VoiceOver or TalkBack [Samsung]); and (8) user-defined alert configurations.

These requirements ensure that the system remains intuitive, adaptable, and accessible across varied contexts.

### Privacy and Security

Privacy and data protection emerged as essential conditions for user trust and adoption. Participants stressed the importance of strict control over personal data, especially given the use of camera-based and location-based technologies. Priority requirements were (1) opt-out options for any nonessential data collection; (2) strict data minimization, limiting processing to navigation-critical information; (3) Al-based identity protection; (4) automatic face blurring in camera streams; (5) clear and strict privacy policies; (6) user-controlled data storage; and (7) robust data protection mechanisms, including encryption and secure authentication.

These safeguards uphold ethical standards and ensure compliance with privacy by design principles. The co-design workshop crystallized this project’s direction by refining user needs into a coherent set of prioritized requirements. Together, the 3 axes of physical accessibility, virtual accessibility, and privacy or security form a comprehensive framework guiding the Edge A-Eye development toward a solution that is (1) inclusive, (2) technically feasible, (3) context-aware, (4) secure, and (5) deeply aligned with the lived experiences of people with visual impairment. Key outputs are organized in Table 4.

**Table 4.** Co-design priorities by axis.

Axis	Key themes	Priority requirements
Physical accessibility	Doors or entrances; signage; object identification; stress; routing; collisions; environmental distractors	Precise door guidance; evacuation plans; camera-based signage reading; product identification; emergency mode; obstacle detection; sound/haptic alerts
Virtual accessibility	Hands-free use; adaptability; instructions; product information; filtering; localization; accessibility; preferences	Voice/haptic control; adjustable volume/speed/contrast; flexible distance units; detailed product info; targeted information modes; estimated time of arrival and floor tracking; screen reader compatibility; customizable alerts
Privacy/security	Geolocation; personal data; camera use; human assistance; guarantees; hyperlinks	Opt-out; data minimization; aliases; facial blurring; strict retention policies; user-controlled storage; safe hyperlink identification

### System Architecture Edge A-Eye

**Figure 5.**
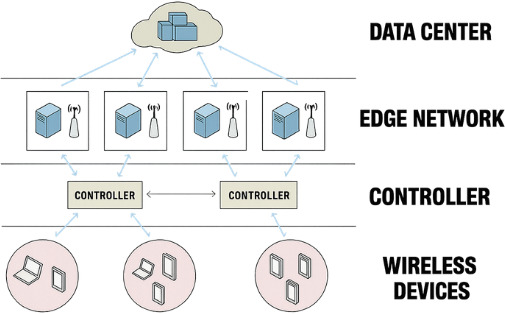
System model of the Edge A-Eye platform.

The system aims to implement an intelligent framework that dynamically composes microservices running on mobile devices, edge, and cloud environments, and then optimally allocates resources to create personalized services for each individual with respect to privacy and security. We will also develop digital-twin models based on the behaviors of people with visual impairment. Figure 5 presents the system model of the Edge A-Eye Platform.

A sophisticated assistive system has been proposed, featuring a mobile app and a cloud-based management platform specifically for individuals with visual impairments. To address navigational challenges faced by those with visual impairment, an advanced automatic navigation system has been developed. This system uses edge computing, federated training, and AI computer vision for object detection and anomaly identification. By integrating these advanced technologies, the system provides visual cues, auditory feedback, and communication features, enhancing path-following accuracy and effective obstacle avoidance. The proposed framework is structured into three layers: (1) cloud computing, (2) edge network, and (3) mobile devices.

When mobile devices lack the computational power to complete tasks within the workflow, they send service requests to edge nodes to obtain the required resources. Controllers are tasked with executing control functions on one or more edge nodes and periodically exchanging messages to maintain synchronization. This method ensures efficient resource management on the edge node.

Server side, we noted three key elements, which are the following: (1) have microservices for the edge-environment to automatically update the map and dataset; (2) frequently store and update the map with obstacles. Additionally, train server-side “anomaly detection” AI model with dataset updated from each device; and (3) AI and training microservices.

Regarding the edge environment, edge computing allows devices in remote locations to process data at the “edge” of the network, either by the device or a local server. Further, when data needs to be processed in the central datacenter, only the most important data is transmitted, thereby minimizing latency. The five key elements were (1) a microservice that is called from people with visual impairment devices, with the people with visual impairment device status and controller signal. It will calculate and draw a full path for people with visual impairment to go on; (2) to update locally the map with patient devices sensor status and Wi-Fi fingerprint; (3) to have received data and produce abnormality detection to warn people with visual impairment and calculate safety; (4) an AI assistant, which can receive raw sound from people with visual impairment device and transfer to text, producing with local large language model, receiving the output it positions to navigate the people with visual impairment; and (5) a federated model, which can share the dataset from a different device and produce it on the Edge A-Eye local server.

### Edge A-Eye System Architecture and User Interaction in a Real-World Setting

**Figure 6.**
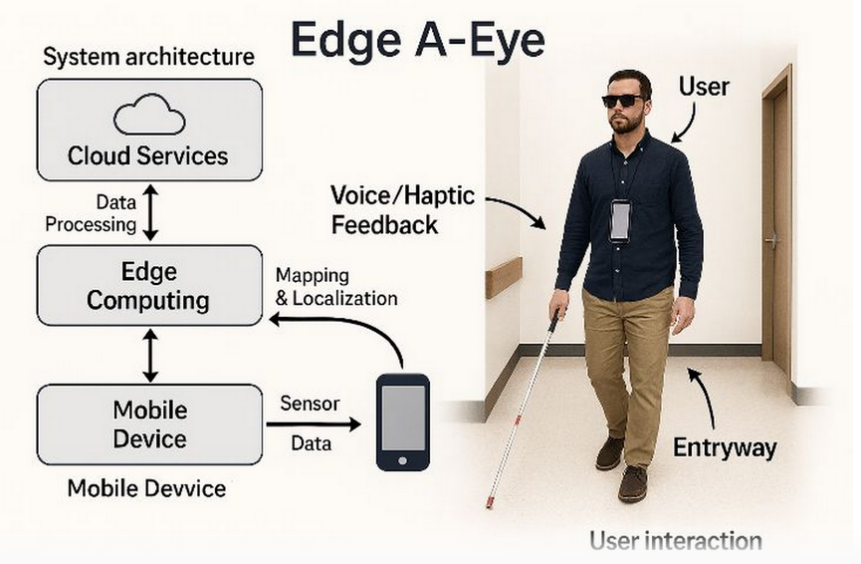
Edge A-Eye system architecture and user interaction in a real-world setting.

It illustrates, first, system architecture (Figure 6, left): cloud services (handles data processing and updates), edge computing (performs mapping and localization tasks), and a mobile device (collects sensor data and communicates with edge or cloud layers). Second, user interaction (Figure 6, right): a person navigating an indoor hallway using a white cane and a smartphone mounted on the chest for hands-free operation, voice or haptic feedback (directions communicated via audio and vibration), and environment (entryway and typical indoor obstacles).

Figure 6 shows the layered architecture (cloud, edge, or mobile device) and illustrates how the user engages with the platform through voice or haptic feedback while navigating an indoor environment.

### Iterative Testing of the Edge A-Eye Platform

Based on the insights from focus groups, interviews and the co-design process, we decided a clinical site was appropriate for real-world testing and the ÉTS building for laboratory testing. Therefore, before testing the Edge A-Eye platform, a detailed mapping and calibration process was conducted at both the ÉTS and LLMRC. This step ensured that navigation scenarios were realistic and adapted to the needs of people with visual impairment. Key outcomes include (1) regarding the identification of critical zones: entrances, reception areas, staircases, elevators, and waiting rooms being mapped as priority navigation points; (2) regarding the contrast and lighting analysis: variations in floor and wall contrasts, lighting intensity, and potential glare sources being documented to optimize visual cues for low-vision users; (3) regarding the spatial marker calibration: distances between key landmarks (doors, corridors, or elevators) being measured and integrated into the navigation algorithm for accurate route planning; (4) regarding the environmental complexity assessment: obstacles such as furniture, signage placement, and noise sources being cataloged to anticipate challenges during navigation; and (5) regarding the scenario definition: two main navigation scenarios being established which were academic setting (ÉTS): navigating from building entrance to designated laboratories; and clinical setting (LLMRC): simulating an optometry visit, including reception, waiting area, consultation room, and optical shop. These findings provided the foundation for precise route planning and adaptive guidance features in the Edge A-Eye platform, ensuring that subsequent usability tests reflected real-world conditions.

A total of 1 vision rehabilitation specialist and people with visual impairment were accompanied by 4 academic and industry partners to evaluate the usability and robustness of the Edge A-Eye platform. These tests were performed on a biweekly basis in the controlled laboratory environment at the ÉTS, with the participation of academic and industry partners alongside a clinician specialized in visual impairment. This stage was critical for assessing ergonomics and robustness, while also identifying technical limitations and guiding necessary adjustments. The collaboration between users and technical experts provided a unique opportunity to refine the solution before moving into real-world testing. For developers, this phase offered several strategic advantages: (1) early detection of technical issues, allowing corrective measures before large-scale deployment; (2) direct feedback from vision rehabilitation specialists and experts, ensuring that design choices were validated against real needs; (3) controlled testing conditions, which facilitated precise measurement of performance and usability; (4) strengthened collaboration with interdisciplinary partners, enabling developers to integrate clinical, industrial, and experiential perspectives into technical refinements; (5) iterative improvement cycle, reducing risks of failure in later stages and accelerating the path toward a stable beta version. This laboratory evaluation demonstrated the essential role of developers in bridging technical feasibility with user experience, ensuring that the Edge A-Eye platform evolved into a reliable, inclusive, and adaptable solution. A video demonstration of this process is available in Multimedia Appendix 7.

### Navigation Performance Across Eight Real-World Tasks

This phase aimed to capture the concrete challenges experienced by people with visual impairment during indoor navigation. A total of 13 people with visual impairment completed 8 structured navigation tasks in clinical indoor environments. Performance varied by lighting, contrast, spatial clarity, and noise levels. These results present explicit, measurable findings that directly inform system requirements. Participants were selected to include a diversity of technological competency, age, and visual status. The demographic characteristics can be seen in Multimedia Appendix 8. Participants were asked to complete the tasks independently using their current mobility tools and devices. The results of participants after completing the 8 sequential tasks (Figure 4) varied considerably across tasks in the success of independent completion of the task, key needs and preferences of the users, as well as the factors in the scene. These results underline the need for the Edge A-Eye platform to have task-dependent capabilities. The results are summarized in Table 5 below.

**Table 5.** Task-based navigation performance and contextual scene factors.

Task (distance; m)	Success rate	Key needs/preferences	Scene factors
Entrance (10)	7/13 (54)	Notification at 6‐8 m; door type; landmarks	High luminance and contrast facilitate entry
Reception (2.5)	8/13 (62)	Immediate notification; positioning	Contrast improves desk detection
Stairs/elevator (7‐13)	11/13, directed (85)	Distance/direction; buttons; handrail	Varies by layout
Waiting area (2.5‐7)	11/13 (85)	Direction to seat; 54% prefer human help	High contrast helpful
Examination room (54)	4/13 (31)	Detailed cues; distance; position; tactile/braille	Fine-grained cues essential
Optical store (18 guided)	—^a^	Assistance preferred (85%); satisfaction 100%	—
Low-vision store (55)	7/13 (54)	Category clarity; accessible payment; directions	Environmental factors cause confusion
Exit (8)	13/13 (100)	Less command guidance; door type; landmarks	High luminance aids orientation

a Not available*.*

A video demonstration of the Edge A-Eye platform iterative testing with an Android (Google LLC) phone, including route planning, voice command, and camera integration at the LLMRC, is available in Multimedia Appendix 9.

### Continuous Dissemination of Results

The dissemination of the results of the Edge A-Eye project was structured around several complementary channels, ensuring scientific, clinical, and intersectoral visibility. Preliminary design and development progress has been shared at scientific conferences (both in vision sciences and engineering), at vision rehabilitation centers, at associations for those with visual impairment, and among interested industry leaders. This dissemination strategy made it possible: (1) to strengthen the scientific legitimacy of this project among peers; (2) to anchor the results in clinical realities and field practices; (3) to foster intersectoral synergies and opportunities for technology transfer; and (4) to support a continuous improvement dynamic by integrating stakeholder feedback at each stage.

### Ongoing Monitoring and Evaluation of the Edge A-Eye Project (May 2024 to December 2025)

The comprehensive evaluation phase applied the TRL framework [40] to monitor technological maturity, identify gaps, and guide improvements. Continuous feedback from real-world testing, clinical partners, and technical teams enabled iterative refinements, ensuring robustness, usability, and long-term viability. This adaptive process-maintained alignment with user needs and operational constraints, paving the way for scalable implementation.

### TRL 1-2: Concept and Feasibility (May to June 2024)

For TRL 1-2, we configured the following: (1) defined user needs through focus groups and interviews; (2) analyzed barriers and facilitators in indoor navigation scenarios; and (3) established a conceptual framework; no technology developed yet.

### TRL 3-4: Preliminary Design (July to November 2024)

For TRL 3-4, we configured the following: (1) conducted co-design workshop (November 1, 2024) to prioritize needs and core features; (2) developed initial wireframes and system architecture; (3) validated feasibility and user alignment; (4) regarding the features and functions included: SITUM (*Smart Indoor Tracking and Indoor Mapping*) indoor mapping for navigation in ÉTS building using Wi-Fi fingerprinting (French only), development of user interface with TalkBack, directional information with gyroscope, obstacle detection, optical character recognition, free of charge, protection of user information (use of test phone); and (5) regarding the features and functions desired: bilingual, accessible with iPhone, customizable directional information, hands-free use, voice activation, accessible interface (text-to-speech and high contrast), haptic door, and obstacle detection.

### TRL 5-6: Controlled Laboratory Testing (December 2024 to March 2025)

For TRL 5-6, we configured the following: (1) performed mapping and calibration at ÉTS and LLMRC; (2) tested prototypes in controlled settings with academic and industry partners; (3) identified technical limitations and applied iterative improvements; (4) regarding the features or functions included: voice activation of navigation, bilingual use (French- and English-language), navigational guidance at 10-second intervals only, activation with physical buttons on phone; (5) regarding the features or functions excluded: gyroscope during route navigation (to permit hands-free use after issues related to manipulation of phone), constant haptic obstacle detection (cognitive overload of testers); and (6) regarding the features or functions desired: prioritize turn-by-turn directions at critical moments, use of camera to read room numbers, rapid door detection near entryways, and use with iOS (Apple Inc).

### TRL 7-8: Real-World Evaluation (July to Sept 2025, Ongoing)

For TRL 7-8, we configured the following: (1) simulated optometry visits at LLMRC with 13 participants; (2) assessed usability, accessibility, and adaptability in authentic contexts; (3) integrated AI features (guidance with optical character recognition [OCR]); (4) regarding the features or functions included: optimized turn-by-turn directions (near approach intersection), integration of floor maps, route planning, use of OCR at destination, and voice activation of destinations at multiple locations; (5) regarding the features or functions excluded: automated integration of OCR (to allow for user to call functions with button or voice); and (6) regarding the features or functions desired: mapping with geomagnetic indoor positioning to improve scalability, use with iOS, customizable voice activation of features, specific detection of objects desired for navigation using YOLO (You Only Look Once; ie, bathrooms, chairs, doors, or desks).

### TRL 9: Scalability and Final Assessment (In Progress)

The results from the previous phases, integrated with laboratory and real-world testing, inform the team as to the next iterations of the Edge A-Eye platform. This structured progression through TRL demonstrates how iterative, user-centered development can transform an initial concept into a robust, inclusive, and scalable AT solution, setting a benchmark for future innovations in indoor navigation for persons with visual impairments. Figure 1 presents ongoing monitoring and evaluation of the Edge A-Eye project (May 2024 to December 2025).

Figure 2 presents the Edge A-Eye TRL radar, summarizing technological maturity and development progress.

### Stakeholder Contributions

In sum, each stage played an irreplaceable role in the co-design of the Edge A-Eye app. People with visual impairment ensured experiential relevance, clinicians guaranteed clinical integration, researchers contributed scientific rigor, and industry partners reinforced technical and economic viability. Together, these stakeholders transformed an idea into an inclusive and sustainable solution.

## Discussion

### Principal Findings

The results of the Edge A-Eye project demonstrate that users desire an indoor navigation platform that is customizable, accessible, and user-friendly. This meant that the platform required accessibility with text-to-speech as well as various screen settings. Additionally, voice activation was of particular interest to the users, as this allowed those with lower technological competencies to use the platform, as well as allowing ease of use with their mobility aids. As only certain types of information are required during the journey, the Edge A-Eye project allowed us to examine and prioritize those pieces of information to avoid cognitive overload while walking in real-world settings. As technical and implementation challenges remain to provide scalability and provide an eventual final assessment of the platform, we continue the co-design process to ensure that it meets users’ needs. This study marks the first time a co-design process for indoor navigation in Canada has been documented. Additionally, our novel approach demonstrates how integrating vision rehabilitation with engineering and computer science can improve user-centered outcomes for indoor navigation.

### Human-Centered Co-Design and Collective Intelligence

The Edge A-Eye project illustrates the transformative impact of human-centered co-design in AT development. By engaging people with visual impairment as co-creators rather than passive testers, this project ensured that lived experiences were embedded at the core of the innovation process. This approach aligns with the six principles of UCD outlined by Ortiz-Escobar et al [17] which emphasize early and continuous user involvement as a foundation for usability, ownership, and long-term adoption. Compliance with ISO 9241-210:2019 strengthened the inclusivity and usability of the Edge A-Eye platform by embedding internationally recognized HCD principles into every stage of development, thereby enhancing adoption potential and reducing abandonment risk [22]. Slattery et al [21] similarly argue that embedding user perspectives across all stages of design enhances both relevance and acceptability, creating solutions that reflect real needs rather than abstract assumptions. Our results confirm that HCD (ISO 9241 210) remains essential but is not sufficient on its own to ensure adoption and scalability in AT [22]. Organizational and regulatory constraints can limit iterative development and hinder large-scale deployment, as illustrated by Chavarria et al [19]. In parallel, the sociomaterial perspective proposed by Rong and Hansopaheluwakan-Edward [24] helps explain why certain constraints (eg, rules, artifacts, and material properties) may become enablers depending on how they are configured. This lens also clarifies our own methodological choices, particularly in the alignment of technical, clinical, and experiential priorities throughout the co-design process. This project also mobilized collective intelligence, defined as the ability of diverse actors to collaborate effectively toward shared goals. By integrating experiential knowledge from people with visual impairment with the expertise of vision researchers, rehabilitation specialists, and developers, Edge A-Eye fostered a shared vision of inclusivity and equity. This convergence of perspectives strengthened the legitimacy of this project and ensured that technical decisions were aligned with social realities. Such collaborative intelligence echoes the developmental evaluation framework by Patton [46], which advocates adaptive learning environments in complex innovation ecosystems. Ultimately, the co-design process transformed diversity into a strategic asset, ensuring that the platform was not only technologically robust but also socially relevant and ethically grounded. This section also highlights the unique contributions of the Edge A-Eye project, including its interdisciplinary co-design approach integrating vision science, engineering, AI, and clinical rehabilitation, and its iterative design process that demonstrably improved usability and user satisfaction compared to conventional solutions.

### Iterative Development and Contextual Evaluation

The success of the Edge A-Eye project was also driven by its reliance on iterative development cycles, which allowed the platform to evolve progressively in response to user feedback. Each prototype, from initial wireframes to functional beta versions, was tested, evaluated, and refined through systematic feedback loops. This iterative agility reflects the principles of inclusive design, where adaptation and responsiveness to real-world contexts are prioritized [17]. By embedding feedback at every stage, the current project reduced risks of failure, avoided costly redesigns, and accelerated the stabilization of the beta version. Equally important was the dual strategy of laboratory-based and real-world evaluations. Controlled laboratory testing provided precise measurements of ergonomics, usability, and technical robustness, while real-world deployments captured the complexities of authentic navigation environments, including noise, crowding, and spatial ambiguity. This combination aligns with best practices in human-computer interaction and accessibility research, which stress the importance of contextual evaluation in producing intuitive and reliable tools [21]. By balancing controlled refinement with ecological validity, the current project ensured that Edge A-Eye was not only technically feasible but also practically usable in diverse environments. This iterative and contextualized approach strengthened both the sustainability and the acceptability of the final product, positioning it as a reliable solution for inclusive indoor navigation.

### Knowledge Dissemination and Continuous Improvement

Active knowledge dissemination was a cornerstone of the Edge A-Eye project, ensuring transparency, collaboration, and ecosystem building around inclusive navigation technologies. Dissemination sessions at ÉTS and the LLMRC, combined with presentations at international scientific conferences, created opportunities for constructive exchanges and systematic feedback. These dialogues validated preliminary results, identified areas for refinement, and opened perspectives for technology transfer into rehabilitation practices. The iterative integration of feedback fostered a feedback-rich environment, enhancing agility, usability, and long-term impact. This process reflects that of the description of the developmental evaluation approach by Patton [46], which embeds continuous learning and adjustment into the innovation cycle. Furthermore, recognition at scientific conferences through awards and international visibility reinforced the current project’s credibility and positioned it as a benchmark in inclusive AT. Ultimately, dissemination was not only about presenting results but about co-constructing the next steps, ensuring that this project remained scientifically rigorous, socially relevant, and sustainable over time.

### Implications From Edge A-Eye Project

Overall, the Edge A-Eye project highlights how co-design, collective intelligence, and iterative development shaped the platform, ensuring that technological robustness was consistently aligned with social relevance. By examining methodological choices, knowledge dissemination strategies, and the ethical commitment to equity and inclusion, the development of the Edge A-Eye platform underscores this project’s dual contribution: advancing scientific understanding of AT design while offering practical recommendations for sustainable, user-driven innovation. Beyond methodological insights, this project provides concrete evidence of impact, linking design principles to measurable outcomes observed during laboratory and real-world evaluations. Concerning other specifications are the following: first, regarding the scientific, this project contributes to the literature on user-centered and co-design methodologies in ATs, specifically in the context of indoor navigation for people with visual impairment. It underscores the value of integrating experiential knowledge by users and rehabilitation specialists into system design to enhance functionality and relevance [31]. Second, methodologically, the current research provides a replicable framework that combines iterative prototyping, participatory design, mixed-context testing, and ongoing evaluation. This approach aligns with best practices in inclusive design and can inform future projects [47]. Third, regarding the ethical and equity, diversity, and inclusion (EDI)–related, by embedding principles of EDI throughout the process, the current project upholds a rights-based approach to innovation. Including users with diverse visual profiles and backgrounds fosters fairer outcomes and helps reduce systemic barriers in digital environments [8,29,30]. Fourth, regarding empowerment, autonomy, and social participation, the current project advances the autonomy of people with visual impairment by positioning them as co-creators rather than passive beneficiaries. This empowerment contributes to increased self-efficacy and social participation, key determinants of well-being and quality of life [6,16,48]. Fifth, regarding the practical and technological, grounding development in actual user contexts ensures a better fit between the platform’s functionalities and daily realities, promoting long-term usability and adoption. Sixth, regarding policy and advocacy, the current study provides evidence supporting the integration of co-design and UCD principles into public innovation strategies and digital accessibility policies. It reinforces the importance of involving end users in shaping the technologies that impact their lives [8,29,30].

### Limitations

Despite the relevant findings from this approach, certain limitations must be acknowledged to contextualize these contributions. The relatively limited number of participants may affect the generalizability of the findings. Including a broader and more diverse sample in terms of age, technological literacy, and types of visual impairment would strengthen future iterations of the platform. Additionally, while this project prioritized user involvement, variations in individual experiences may result in different levels of usability or satisfaction across user groups. The technical ecosystem also presented limitations: compatibility issues with different devices and ATs could restrict access for some potential users. Addressing these interoperability challenges is crucial for ensuring broader implementation and adoption. Understanding these strengths and limitations offers valuable guidance for refining the platform, scaling the innovation, and informing similar projects within the field of inclusive technology and assistive navigation.

### Conclusions

This study demonstrates that a sustained participatory co-design process can significantly strengthen the development of indoor navigation technologies for people with visual impairments. Through iterative stages, the integration of user, clinical, engineering, and industry perspectives led to meaningful refinements of the Edge A-Eye platform, including improvements in navigation clarity, interpretation of environmental cues, hands-free interaction, and optimization for iPhone compatibility. User feedback directly informed several critical design decisions, such as reducing unnecessary obstacle alerts, removing gyroscope-dependent interactions due to usability and battery constraints, and addressing challenges related to digital accessibility and varying levels of technological literacy. These findings confirm that co-design not only enhances usability but also ensures alignment with real-world mobility needs, safety considerations, and user expectations. Methodologically, this work contributes a replicable, multiphase co-design framework tailored to the rehabilitation and AT context in Canada. These technological advancements translate into tangible benefits for people with visual impairment, including greater independence, improved safety, and smoother navigation in daily life, reinforcing the social impact of the Edge A-Eye platform. Beyond methodological insights, the Edge A-Eye platform demonstrated measurable improvements during iterative testing. Laboratory and real-world evaluations confirmed continuous enhancement of navigation clarity and environmental interpretation. Across two indoor sites, guidance performance improved with optimized turn-by-turn directions and reduced cognitive load. User satisfaction was high, with participants reporting smoother navigation and fewer directional errors compared to initial prototypes. These findings provide concrete evidence of technological progress and reinforce the platform’s potential for real-world adoption.

Recommendations grounded in study findings are the following: (1) prioritize scenario-specific route guidance and minimize cognitive load to prevent information overload, (2) maintain accessibility features that accommodate diverse technological literacy levels and common mobile accessibility practices, and (3) continue iterative refinement to ensure usability and safety across varied indoor environments.

Broader future research directions are the following: (1) conduct long-term evaluations in diverse real-world settings with larger and more heterogeneous people with visual impairment populations to validate adoption and performance stability, (2) explore the integration of emerging technologies (eg, augmented reality, advanced environmental mapping, and multimodal feedback) to enhance navigation efficiency and situational awareness; (3) develop strategies for scalability and affordability to ensure equitable access across socioeconomic and linguistic groups; and (4) promote knowledge sharing and interdisciplinary collaboration to sustain innovation and inclusivity in AT development.

By embedding these priorities, future iterations of Edge A-Eye can advance autonomy, confidence, and day-to-day safety for people with visual impairment, while contributing to a broader framework for inclusive, context-aware technological innovation.

## Supplementary material

10.2196/81347 Multimedia Appendix 1 Semistructured interview guide for the Edge-A-Eye project.

10.2196/81347 Multimedia Appendix 2 Co-design workshop.

10.2196/81347 Multimedia Appendix 3 Questionnaire for selection of participants for Edge A-Eye.

10.2196/81347 Multimedia Appendix 4 Prescenario questionnaire.

10.2196/81347 Multimedia Appendix 5 Clinical walkthrough questionnaire (barriers and facilitators).

10.2196/81347 Multimedia Appendix 6 Postscenario questionnaire.

10.2196/81347 Multimedia Appendix 7 Video on Edge A-Eye app.

10.2196/81347 Multimedia Appendix 8 Participant demographics for real-world testing (n=13).

10.2196/81347 Multimedia Appendix 9 Video on navigation.

10.2196/81347 Checklist 1 COREQ checklist.

## Abbreviations

AI: artificial intelligence
ASAMM: Association des Sports Pour Aveugles du Montréal Métropolitain
AT: assistive technology
CERC: Clinical Research Ethics Committee
CNIB: Canadian National Institute for the Blind
COREQ: Consolidated Criteria for Reporting Qualitative Research
CRIR: Centre de Recherche Interdisciplinaire en Réadaptation du Montréal Métropolitain
EDI: equity, diversity, and inclusion
HCD: human‑centered design
INLB: Institut Nazareth et Louis-Braille
ISO: International Organization for Standardization
IVADO: Institut de Valorisation des Données
LLMRC: Lethbridge-Layton-Mackay Rehabilitation Centre
NAT: navigation‑based assistive technology
O&M: orientation and mobility
OCR: optical character recognition
RAAMM: Regroupement des Aveugles et Amblyopes du Montréal Métropolitain
TA: TA
TRL: technology readiness level
UCD: user‑centered design
YOLO: You Only Look Once
ÉTS: École de Technologie Supérieure
