# Supplementary material for: Indoor Navigation for People With Visual Impairment in Canada: Participatory Co-Design and Interdisciplinary Study of the Edge A-Eye Platform
Source: JMIR Rehabil Assist Technol. 2026 Jul 31;13:e81347. doi: 10.2196/81347 (PMC13427077; doi:10.2196/81347)
Supplement: Multimedia Appendix 1 — Semistructured interview guide for the Edge-A-Eye project. [file rehab-v13-e81347-s001.docx]

A Semi structured interview will be conducted virtually via zoom for approximately 120 minutes. We are anticipating 5 participants with a diversity of age, levels of visual impairments, and travel habits, based on the intake questionnaire in each focus group. We will start with the consent for audio recording of the meeting which will be followed by quick explanation of the structure of the meeting (e.g., informing them about number of questions and how much time will be given to each question). We will approximately give 10 minutes to each question and each participant will take turns to answer the question. The rules of engagement in discussion will be provided to the participants at the start of the session (e.g. taking turns, etc.). Additionally, we will use prompting questions to guide them in case the participant does not respond or takes too long to respond. The participant may raise their virtual hand if they have any queries or confusions.

ICE-BREAKER: What is your favorite smartphone app, and why?

The members of the focus group will imagine going through each of the following scenarios independently (even if they do not typically do this on their own) and respond to the questions following each subtask involved in the scenario. Additional open-ended questions follow each scenario.

Clinic appointment scenario

1. You are going to your regular eye appointment
   - What makes it easier or more difficult to find the clinic? How does tech help?
2. You are entering the clinic
   - What makes it more difficult or easier to enter? How does tech help?
3. You are going to the reception desk
   - What makes it easier or more difficult? How does tech help?
4. You are going to the waiting area and sitting down
   - What makes it more easy or difficult? How does tech help?
5. It’s your turn to see the specialist, so you are headed to the appropriate exam room
   - What makes it more easy or difficult? How does tech help?
6. You are entering the exam room
   - What makes it more easy or difficult? How does tech help?
7. You are interacting with your eye care professional during the visit
   - What makes it more easy or difficult? How does tech help?
8. You are leaving the exam room and headed to the reception
   - What makes it more easy or difficult? How does tech help?
9. You are checking out and/or paying for the visit
   - What makes it more easy or difficult? How does tech help?
10. You are leaving the clinic
    - What makes it more easy or difficult? How does tech help?

What are the strengths/weaknesses of currently available technology that you use in this scenario?

What are the gaps of currently available technology?

What would be included in your ideal technology for this scenario?

Shopping scenario

1. You are going to your grocery store
   - What makes it easier or more difficult to find it? How does tech help?
2. You are entering the store
   - What makes it more difficult or easier to enter? How does tech help?
3. You are getting a basket
   - What makes it easier or more difficult? How does tech help?
4. You are figuring out the layout of the store
   - What makes it more easy or difficult? How does tech help?
5. You are going to the aisles that interest you
   - What makes it more easy or difficult? How does tech help?
6. You want to identify the items to make the right choice
   - What makes it more easy or difficult? How does tech help?
7. You are checking the prices and take advantage of discounts
   - What makes it more easy or difficult? How does tech help?
8. You are going to the checkout counter
   - What makes it more easy or difficult? How does tech help?
9. You are paying for your items
   - What makes it more easy or difficult? How does tech help?
10. You are leaving the store
    - What makes it more easy or difficult? How does tech help?

What are the strengths/weaknesses of currently available technology that you use in this scenario?

What are the gaps of currently available technology?

What would be included in your ideal technology for this scenario?
