# Supplementary material for: Indoor Navigation for People With Visual Impairment in Canada: Participatory Co-Design and Interdisciplinary Study of the Edge A-Eye Platform
Source: JMIR Rehabil Assist Technol. 2026 Jul 31;13:e81347. doi: 10.2196/81347 (PMC13427077; doi:10.2196/81347)
Supplement: Multimedia Appendix 2 — Co-design workshop. [file rehab-v13-e81347-s002.docx]

|  |  |  |  |  |  |
| --- | --- | --- | --- | --- | --- |
|  |  |  |  |  |  |
| **Documenting user needs and requirements for assistive indoor navigation platform Edge A-Eye** | | | | |  |
|  |  |  |  |  |  |
| The purpose of the co-design workshop is to better understand the needs and priorities of users with visual impairments for the  creation, design, and development of a user-friendly and accessible indoor navigation platform | | | | | |
|  |  |  |  |  |  |
| There are 3 separate modules intended to stimulate discussion, with several items highlighted by the research team. | | | | | |
| Each team is comprised of members of industry, clinical research, engineering research, and a member of the community with a  visual impairment | | | | | |
|  |  |  |  |  |  |
| **Physical accessibility** |  |  |  |  |  |
| Area of interest | Question |  |  |  | Priority 4 |
| Doors/entryways | What challenges do you face with entryways and doors while navigating? |  | | |  |
| Signage | What challenges do you face reading signs while navigating? |  | | | |
| Object identification | What difficulties do you have finding or identifying objects of interest while indoors (outside the home)? |  | | | |
| Stress/frustration | Which situations cause you the most stress or frustration when navigating indoor spaces? |  | | |  |
| Route planning | Which strategies are most successful for you when planning new routes indoors? |  | | | |
| Collisions/accidents | What issues do you have around hitting or missing objects or people that may be of concern for your safety? |  | | | |
| Environmenal distractors | Which things is your environment distract you the most from arriving at your destination? |  | | | |
|  |  |  |  |  |  |
| **Virtual accessibility** |  |  |  |  |  |
| Area of interest | Question |  |  |  | Priority 4 |
| Hands Free Use | What methods work best for you to allow the use of a mobile platform (i.e. cell phone) without holding it? Or which methods would you like to use? |  | | | |
| Environmental adaptability (lighting, noise) | How would an app best adjust to the environment while navigating to improve your navigation? |  | | | |
| Navigation instructions | How would you like the navigation instructions presented to you? |  | |  |  |
| Item information (labels, expiration) | Which type of information should be made more accessible to you when purchasing an item when shopping? |  | | | |
| Information filtering | Which information is unnecessary and should be filtered out while you are navigating? |  | | | |
| Location Tracking | What real-time information do you need about your route and destination while you are navigating? |  | | |  |
| Accessibility settings | Which settings do you use to enhance your mobile experience? Which is your preferred method? |  | |  |  |
| Notification Preference | What are your preferences regarding the timing and method of alerts from the platform while you are navigating? |  | | | |
|  |  |  |  |  |  |
| **Privacy and security concerns** |  |  |  |  |  |
| Area of interest | Question |  |  |  | Priority 4 |
| Geolocation | What are your biggest concerns related to sharing your current position? |  | | | |
| Personal data | What are your biggest concerns related to the sharing of your personal data? |  | | |  |
| Use of camera | What are your biggest concerns about the use of a camera on a mobile device? |  | | | |
| Human assistant | What concerns do you have about sharing your personal information with another person over the phone? |  | | | |
| Warranties | What type of warranties or assurances would you need to feel secure in using the platform |  | | | |
| Hyperlinks | What are your biggest concerns related to the platform containing links to external sites? |  | | | |
|  |  |  |  |  |  |
