# Supplementary material for: Indoor Navigation for People With Visual Impairment in Canada: Participatory Co-Design and Interdisciplinary Study of the Edge A-Eye Platform
Source: JMIR Rehabil Assist Technol. 2026 Jul 31;13:e81347. doi: 10.2196/81347 (PMC13427077; doi:10.2196/81347)
Supplement: Multimedia Appendix 4 — Prescenario questionnaire. [file rehab-v13-e81347-s004.docx]

Length: Approximately 5-10 minutes

In the form of a discussion with a member of the research team

Just before starting the assigned scenario

**__________________________**

**Participant contact information**

First name:

Last name:

Telephone number:

Email address:

**__________________________**

**Scenario for this participant:**

__Going to an appointment at a clinic (eg. optometrist)

__Shopping

**__________________________**

1. How often do you perform this type of scenario independently in an unfamiliar environment? (eg. weekly, occasionally, never)

2. What mobility aid (other than the smartphone) do you plan to use during this scenario? (eg. white cane, guide dog, optical aid)

3. On your smartphone, what accessibility settings will be enabled in this scenario? (eg. screen reader, zoon, etc.)

4. In anticipating the scenario, which steps seem to be the most difficult in your opinion?

5. Are there any mobile apps that you are thinking of using during some of the steps in this scenario? If so, which ones?

6. If you need to listen to the sound of your phone during the scenario, how will you do it? (eg. built-in speaker, headphones, hearing aids)

7. In general, how would you rate your level of confidence in performing this scenario?
