# Supplementary material for: Indoor Navigation for People With Visual Impairment in Canada: Participatory Co-Design and Interdisciplinary Study of the Edge A-Eye Platform
Source: JMIR Rehabil Assist Technol. 2026 Jul 31;13:e81347. doi: 10.2196/81347 (PMC13427077; doi:10.2196/81347)
Supplement: Multimedia Appendix 5 — Clinical walkthrough questionnaire (barriers and facilitators). [file rehab-v13-e81347-s005.docx]

**Task-Specific Questions (Tasks numbered 1 to 8)**

Each task questioning will follow a similar structure:

a. What difficulties did you encounter in completing this step/task?

b. What facilitators made this task easier for you?

c. Are there any mobile applications that could assist or make this step easier to navigate? If yes, which ones? And How?

1. **Entering the Clinic – Finding the Main Entrance Door**

a. What difficulties did you encounter in completing this step/task?

b. What facilitators made this task easier for you?

c. Are there any mobile applications that could assist or make this step easier to navigate? If yes, which ones? And How?

**2.Navigating to the Reception Desk**

a. What difficulties did you encounter in completing this step/task?

b. What facilitators made this task easier for you?

c. Are there any mobile applications that could assist or make this step easier to navigate? If yes, which ones? And How?

1. **Navigating from the first floor to the third floor(Stairs or Elevator)**

a. What difficulties did you encounter in completing this step/task?

b. What facilitators made this task easier for you?

c. Are there any mobile applications that could assist or make this step easier to navigate? If yes, which ones? And how?

**4.Finding the Waiting Area on the Third Floor**

a. What difficulties did you encounter in completing this step/task?

b. What facilitators made this task easier for you?

c. Are there any mobile applications that could assist or make this step easier to navigate? If yes, which ones? And How?

1. **Finding the Appointment Room**

a. What difficulties did you encounter in completing this step/task?

b. What facilitators made this task easier for you?

c. Are there any mobile applications that could assist or make this step easier to navigate? If yes, which ones? And How?

1. **Moving from the Consultation Room to the Optical Room on the same floor**

a. What difficulties did you encounter in completing this step/task?

b. What facilitators made this task easier for you?

c. Are there any mobile applications that could assist or make this step easier to navigate? If yes, which ones? And How?

1. **Traveling to the Low Vision Boutique on the First floor**

a. What difficulties did you encounter in completing this step/task?

b. What facilitators made this task easier for you?

c. Are there any mobile applications that could assist or make this step easier to navigate? If yes, which ones? And How?

1. **Finding the exit of the building**

a. What difficulties did you encounter in completing this step/task?

b. What facilitators made this task/step easier for you?

c. Are there any mobile applications that could assist or make this step easier to navigate? If yes, which ones? And How?
