# Supplementary material for: Indoor Navigation for People With Visual Impairment in Canada: Participatory Co-Design and Interdisciplinary Study of the Edge A-Eye Platform
Source: JMIR Rehabil Assist Technol. 2026 Jul 31;13:e81347. doi: 10.2196/81347 (PMC13427077; doi:10.2196/81347)
Supplement: Multimedia Appendix 6 — Postscenario questionnaire. [file rehab-v13-e81347-s006.docx]

Length: Approximately 5-10 minutes

In the form of a discussion with a member of the research team

Just after finishing the assigned scenario

**__________________________**

**Participant contact information**

First name:

Last name:

Telephone number:

Email address:

**__________________________**

**Scenario for this participant:**

__Going to an appointment at a clinic (eg. optometrist)

__Shopping

**__________________________**

1. In your opinion, did you manage to complete all the stops in this scenario?

2. What were the difficulties encountered?

3. What strategies did you use to overcome these difficulties? (e.g. use of mobile application, asking for help, etc.)

4. Did you use any mobile application(s) while working on the scenario and why? If yes, which ones?

5. Did the applications used, if any, result in the desired information? Explain your answer.

6. During which stage of the scenario did you find the smartphone to be most useful? Explain your answer.

7. In your opinion, are there any need(s) identified in the scenario that are not adequately addressed by current technologies?
