# Supplementary material for: Indoor Navigation for People With Visual Impairment in Canada: Participatory Co-Design and Interdisciplinary Study of the Edge A-Eye Platform
Source: JMIR Rehabil Assist Technol. 2026 Jul 31;13:e81347. doi: 10.2196/81347 (PMC13427077; doi:10.2196/81347)
Supplement: Multimedia Appendix 8 — Participant demographics for real-world testing (n=13). [file rehab-v13-e81347-s008.docx]

| **Code** | **Gender** | **Age** | **Ethnicity** | **Language** | **Age at diagnosis** | **Visual Diagnosis** | **Visual status** | **Mobility aids** | **OS** |
| --- | --- | --- | --- | --- | --- | --- | --- | --- | --- |
| AF40 | M | 64 | Asian | English | 15 | Glaucoma | LV | White Cane | iPhone |
| AF37 | F | 30 | Latino | English | Birth | Premature retinopathy, Retinal detachment | CB | White cane | iPhone |
| AF31 | F | 34 | Caucasian | English | Birth | Disease of retina | CB | White cane | iPhone |
| F37 | M | 73 | Caucasian | French | 30 | Retinitis Pigmentosa | CB | White cane | iPhone |
| F36 | F | 61 | Caucasian | French | Birth | Optic nerve atrophy, Congenital macula atrophy | LV | None | Android |
| F43 | M | 61 | African | French | Birth | Ocular albinism | LV | None | iPhone |
| AF38 | M | 67 | Latino | English | 6 years old | Chorioretinitis | LV | White cane | iPhone |
| F41 | M | 49 | Caucasian | French | 35 years | Retina detachment | LV | White cane | IPhone |
| AF39 | M | 32 | Asian | English | 6 months | Glaucoma | CB | White cane | IPhone |
| F46 | M | 62 | Caucasian | French | 40 years | N/A | CB | White Cane | IPhone |
| AF42 | M | 63 | Canadian | English | 51 years | Optic Nephritis | LV | White Cane | IPhone |
| AF43 | M | 30 | African | English | 10 years | Starrgadt Disease | LV | N/A | Android |
| AF 44 | M | 41 | Latino | English | 3 months | Choriorétinites | CB | White Cane | IPhone |
